# Clinical factors influencing knowledge and self‐care practice among adults with type 2 diabetes mellitus

**DOI:** 10.1002/nop2.1506

**Published:** 2022-12-04

**Authors:** Richard Adongo Afaya, Victoria Bam, Alberta Yemotsoo Lomotey, Agani Afaya

**Affiliations:** ^1^ Department of Midwifery and Women's Health University for Development Studies Tamale Ghana; ^2^ Department of Nursing Kwame Nkrumah University of Science and Technology Kumasi Ghana; ^3^ Department of Nursing, School of Nursing and Midwifery University of Health and Allied Sciences Ho Ghana; ^4^ College of Nursing Yonsei University Seoul South Korea

**Keywords:** clinical factors, diabetes mellitus, influencing factors, knowledge, self‐care practice, type 2 diabetes mellitus

## Abstract

**Aim:**

The objective of the study was to determine the clinical factors associated with knowledge and self‐care practice among adults living with type 2 diabetes mellitus.

**Design:**

Descriptive cross‐sectional design.

**Methods:**

A convenience sample of 330 participants was recruited over 3‐months in 2018 and data were collected using a structured instrument.

**Results:**

Participants on insulin treatment modality had four times higher odds of knowledge on diabetes (*B* = 4.17, *p* = 0.023) while those on combined therapy (both oral hypoglycaemic agent and insulin) had 7.26 times higher odds of knowledge (*B* = 7.26, *p* < 0.001). Participants without medically confirmed diabetic complications had 3.66 higher odds of knowledge of diabetes (*B* = 3.66, *p* = 0.002). Participants on insulin treatment modality had a 1.4‐fold higher odds of self‐care practice (*B* = 1.4, *p* = 0.028). It was revealed that participants with hypertension and diabetic foot had lower odds of self‐care practice (*B* = −1.13, *p* = 0.021).

**Conclusion:**

In particular, participants who were on insulin and combined therapy (tablet and insulin) had higher knowledge and better self‐care practice. Self‐care was significantly influenced among those with, than those without diabetic foot and hypertension as complications.

## 1 INTRODUCTION

The increasing prevalence of diabetes mellitus (DM) imposes a huge economic burden all over the world, particularly in sub‐Saharan Africa where there are limited resources to manage the disease and its impact (Herrington et al., 2018; Islam et al., 2015). In 2017, it was estimated that 425 million people had diabetes worldwide with a corresponding global prevalence of 8.8% (International Diabetes Federation Report [IDF], 2017). The rates of DM have risen. An overwhelming 80% reside in low‐ and middle‐income countries (International Diabetes Federation, 2013) that are not well equipped to tackle this emerging catastrophe (International Diabetes Federation, 2013; Islam et al., 2015; Sarfo‐Kantanka et al., 2016). In sub‐Saharan Africa including Ghana, type 2 diabetes mellitus (T2DM) accounts for most cases. The International Diabetes Federation's report indicated that Ghana had a raw national prevalence of 3.6% in 2017 (IDF, 2017). Diabetes prevalence studies done in Ghana vary across different regions ranging from 6.2% to 9.2% (Asamoah‐Boaheng et al., 2019; Cook‐Huynh et al., 2012). A recent comprehensive systematic review and meta‐analysis established a high overall diabetes prevalence rate of 6.4% among the Ghanaian adult population (Asamoah‐Boaheng et al., 2019). The surge in the prevalence of T2DM is alarming as Ghana is faced with inadequate healthcare facilities, an inadequate number of skilled health personnel and limited resources, and all these significantly affect the healthcare system and the economy (Mogre et al., 2017; World Health Organization, 2016).

Uncontrolled T2DM is associated with many irreversible complications affecting both micro‐vascular (nephropathy, retinopathy, and neuropathy) and macro‐vascular (heart diseases) systems of the body (Karaoui et al., 2018). These complications have led to increased disability, high mortality rates, decreased quality of life and severe economic burden for every society (IDF, 2015).

Having adequate knowledge of T2DM is very crucial in diabetes management. In order to reach optimal glycaemic targets and attenuate complications, people with T2DM should have adequate knowledge of diabetes to enhance adherence to self‐care activities such as diet, exercise, self‐monitoring of blood glucose, foot care and taking of medication (Gautam et al., 2015; Kassahun et al., 2016). Self‐care is the most important facet of diabetes management and studies have shown that it improves glycaemic control, reduces healthcare expenditure, prevents complications and enhances quality of life (Iregbu & Iregbu, 2016; Jannoo & Mamode Khan, 2018). However, for people with T2DM, self‐care which is made up of 98% of diabetes management is very demanding, multifaceted and intricate (Chali et al., 2018; Jannoo & Mamode Khan, 2018). Good self‐care practice is required to keep the disease under control (Abate et al., 2018) and evidence on the knowledge and self‐care practice of adults with T2DM will provide vital insight for developing well‐targeted preventive strategies to reduce the burden of diabetes (Herath et al., 2017; Karaoui et al., 2018).

Various studies have found clinical factors such as duration of diabetes (Alhaik et al., 2019; D'Souza et al., 2017) and treatment type (Abate et al., 2018; Alhaik et al., 2019) to have an influence on knowledge and self‐care practice. Moreover, patients who have suffered from complications such as amputation, impaired vision, may have challenges with self‐care (Sparring et al., 2013). Healthcare providers' identification of factors associated with knowledge and self‐care practice in people with T2DM will inform decisions regarding specific areas and populations of patients that will require specific attention and support. Though studies have been conducted on diabetes knowledge and practice in developing countries (Karaoui et al., 2018), the available literature focusing on clinical factors associated with knowledge and self‐care practice of diabetes is limited. It was against this background that we sought to determine clinical factors influencing knowledge and self‐care practice of adults with T2DM in government hospitals in Tamale, Northern Ghana. Thus, the research question is, what are the clinical factors associated with knowledge and self‐care practice among adults living with T2DM in northern Ghana?

### 1.1 Objectives of the study

- To determine clinical factors influencing knowledge and self‐care practice.
- To evaluate the association between diabetic complications and self‐care practice.

## 2 METHODS

### 2.1 Study design and setting

This was a multicentre analytical cross‐sectional study conducted at three government hospitals in Tamale, the capital of the northern region of Ghana. The Tamale metropolis has a total population of 233,252 with 80.8% of the inhabitants being urban dwellers (Ghana Statistical Service [GSS], 2014). People from all parts of Ghana are living and working in the city. The hospitals have diabetes clinics providing routine outpatient services for persons with diabetes from both urban and rural settings. The clinics are largely patronized by urban dwellers.

### 2.2 Study population

All persons with T2DM who presented to the diabetes clinics of the selected hospitals for routine visits constituted the target population for the study. The patients were eligible to participate if they were aged 30 years or more, had T2DM for at least 1 year and were registered with the particular hospital. Type 1 diabetes mellitus patients and those with some form of mental incapacity were excluded from the study.

### 2.3 Outcome measures

The main outcome variables in this study were diabetes knowledge and self‐care practice. Knowledge was assessed with the Diabetes knowledge test developed by scholars from the University of Michigan (Fitzgerald et al., 1998). Self‐care practice was measured using the revised version of the Summary of Diabetes Self‐Care Activities (SDSCA) questionnaire (Toobert et al., 2000).

### 2.4 Explanatory variables

Clinical characteristics including family history, blood pressure, body mass index, waist circumference, smoking, duration of diabetes and diabetic complication were the main explanatory variables. Family history of diabetes was assessed using a question; ‘Has any of your family member (mother or father) had diabetes?’ Participants were also asked if they had ever smoked in their lifetime. Participants were asked whether they had diabetic complications and this was subsequently confirmed by chart review. Socio‐demographic characteristics (age, gender, education status, occupation, marital status, religion and family support) were excluded as explanatory variables.

### 2.5 Sampling and sample size determination

Non‐probability convenience sampling technique was employed to recruit 360 participants from September to November of 2018. The sample size for the survey was determined using the Yamane formula for sample size calculation (Yamane, 1967).

Using an estimated population of 1800 cases based on the institutions' registry, a sample size of 327 participants was needed for this study. The possibility of making a type I error was estimated at 5% with a 95% confidence interval. A 10% non‐response rate was calculated and added to the required sample size, thus, increasing it to 360.

### 2.6 Study instrument

The study instrument was a structured questionnaire and consisted of four sections (A, B, C and D). The first section (Section A) had demographic characteristics including age, sex, marital status, level of income, level of education, occupation, place of residence and family history of diabetes. Section B consisted of clinical characteristics such as duration of diabetes, treatment type, family history, weight, height, body mass index and blood pressure.

Section C assessed diabetes‐related knowledge among participants using the Diabetes Knowledge Test (DKT) (Fitzgerald et al., 1998). This is a 23‐item tool with questions related to diagnosis, signs and symptoms, causes, risk factors, prevention and complications of diabetes. Each question had one correct answer from two answer choices. The answer choices for each question were ‘Yes’ and ‘No’. The internal consistency measured by Cronbach's alpha for the items of the knowledge was 0.69 in the present study.

Section D consisted of respondents' practice of self‐management activities. The patients' self‐care practices were measured by the revised version of the Summary of Diabetes Self‐Care Activities (SDSCA) questionnaire (Toobert et al., 2000). The SDSCA is a valid and reliable tool popularly used in diabetes management with a reliability of 0.74 (Vincent et al., 2008). It was used to assess respondents' self‐reported frequency of adherence to self‐care practice. The SDSCA had five important subscales including diet (general and specific), exercise, self‐monitoring of blood glucose, foot care and not smoking cigarettes. Respondents were required to rate the number of days they performed a specific self‐management practice during the last 7 days. The scale ranged from 0 to 7 with greater scores corresponding to better self‐care.

### 2.7 Data collection procedure

Three registered nurses were trained as research assistants to ensure uniformity in the administration of the questionnaire. All research assistants were trained on the study protocol, the aim and objectives of the study, how to take anthropometric measures and blood pressure, how to approach and recruit participants and how to translate questions to participants during the data collection without revealing answers. Data were collected at the outpatient diabetes clinics weekly on days scheduled by the health facilities to provide routine care to patients. They approached patients who presented to the health facilities for their routine clinic visits and invited them to participate in the study. All patients who volunteered to take part in the study were screened for eligibility. The purpose of the study was explained and written informed consent was obtained from participants before questionnaire administration. The questionnaires were paper‐based and self‐administered to participants who were able to read and write in English. For those who were unable to read or write in English, trained research assistants assisted them to complete the questionnaire by translating the questions into their respective local dialects verbally. It took an average of about 30 min to complete a questionnaire.

### 2.8 Anthropometric measures

The patients' weights were taken with the patients wearing light clothing and without their shoes/sandals using an electronic scale produced by Seca. Height was also taken and body mass index (BMI) was calculated. Weight was measured to the nearest kilogram and height in centimetres. In calculating BMI, height was converted to metres. The BMI was computed in accordance with standard guidelines by WHO and categorized as underweight, normal weight, overweight and obese (WHO, 2000).

Waist circumference (WC) was assessed with the patients in an upright position and measurement was taken midway between the inferior angle of the ribs and the supra‐iliac crest (WHO, 2008) to the nearest 1 cm using a non‐stretchable fibre‐glass measuring tape (Butterfly, China). Abdominal obesity was measured as a waist circumference >102 cm in men and >88 cm in women based on the WHO guidelines (WHO, 2008).

### 2.9 Data analysis

Out of the 360 participants that were invited, 330 of them consented to participate, resulting in 91.7% response rate. The 30 who declined participation expressed their disinterest in the study. The research assistants glanced through all sections of the questionnaires to ensure their completeness. Subsequently, there were no missing data in the collected questionnaires. All statistical analysis was done with the Statistical Package for Social Sciences (SPSS) version 25.0. Descriptive statistics were used to describe the demographic and clinical characteristics of the respondents. Multivariable logistic regression was done to predict the influencing clinical factors of knowledge and self‐care practice. Statistical significance was considered at a *p* value of less than 0.05.

### 2.10 Reliability and validity

The opinion of six experts with extensive experiences in the disciplines of nursing and medicine was sought to establish the face and content validity of the items. The experts assessed the face and content validity of each item on the questionnaires by evaluating their clarity, comprehensibility, relevance, simplicity and grammatical construction. After the validation process, a pretest was done among 25 participants whose data were not included in the analysis. The Cronbach alpha coefficient for the instrument was 0.78, showing good internal consistency.

### 2.11 Ethical considerations

Approval for this study was granted by the Committee on Human Research, Publication and Ethics of the Kwame Nkrumah University of Science and Technology/Komfo Anokye Teaching Hospital (CHRPE/AP/576/18). Permission was sought from the heads of the hospitals to conduct the study in their facilities. The purpose and objectives of the study were explained to respondents and written informed consent was obtained before data collection. The patients were informed that participation is voluntary and that they can opt‐out of the study at any time without any consequences to them. Respondents were assured of confidentiality and that no personal identifiers will be used in the questionnaire.

## 3 RESULTS

### 3.1 Socio‐demographic characteristics of participants

Table 1 shows the socio‐demographic characteristics of participants. The mean age was 57.5 ± 11.8 (range = 30–91) years and females formed about two‐thirds, 225 (68.2%) of the study population. More than half, 200 (60.6%) of the respondents had no formal education and 143 (43.3%) of them were self‐employed while 106 (32.1%) were unemployed.

**TABLE 1:** Socio‐demographic characteristics of participants

Variable	Frequency (%)
Age	
Mean age + SD	57.5 + 11.8
Gender	
Male	105 (31.8)
Female	225 (68.2)
Education	
Tertiary	50 (15.1)
Senior high school	33 (10.0)
Junior high school	24 (7.3)
Primary school	23 (7.0)
No formal education	200 (60.6)
Occupation	
Private sector employment	25 (7.6)
Public sector employment	56 (17.0)
Self‐employment	143 (43.3)
No employment	106 (32.1)
Marital status	
Single	16 (4.8)
Married	223 (67.6)
Divorced	29 (8.8)
Widowed	62 (18.8)
Family support	
Yes	265 (80.3)
No	65 (19.7)
Religion	
Christian	84 (25.5)
Muslim	246 (74.5)

### 3.2 Clinical characteristics of participants

Table 2 shows the clinical characteristics of respondents. Almost three‐quarters, 247 (74.8%) of the patients were on oral hypoglycaemic treatment while a tenth, 33 (10%) were taking insulin injections and the remaining 50 (15.2%) were on both oral hypoglycaemic agents and insulin. A large proportion, 130 (39.4%) had medically confirmed diabetic complications including hypertension, foot ulcer, retinopathy, kidney disease and neuropathy. The mean BMI was 26.4 ± 6.3 and a substantial proportion was not within the normal weight range. Thus, obese, overweight and underweight were 81 (24.6%), 107 (32.4%) and 25 (7.6%) respectively. About two‐thirds, 227 (68.8%) of the respondents were abdominally obese.

**TABLE 2:** Clinical characteristics of participants

Characteristics	Frequency (%)
Family History of diabetes	
Yes	130 (39.4)
No	130 (39.4)
Do not Know	70 (21.2)
Duration of Diabetes	
1–3 years	126 (38.2)
4–6 years	99 (30.0)
7–9 years	45 (13.6)
10+ years	60 (18.2)
Type of treatment	
Oral hypoglycaemic	247 (74.8)
Insulin	33 (10.0)
Both oral and Insulin	50 (15.2)
Medically confirmed diabetic complications	
Yes	130 (39.4)
No	193 (58.5)
Not sure	7 (2.1)
Body Mass Index (BMI)	
Mean BMI + SD	**26.4 + 6.3**
Obese	81 (24.6)
Overweight	107 (32.4)
Normal weight	117 (35.4)
Underweight	25 (7.6)
Waist Circumference	
Mean WC + SD	
Abdominally obese	227 (68.8)
No abdominal obesity	103 (31.2)

### 3.3 Association between clinical characteristics and knowledge of participants

In Table 3, multivariable regression analysis was used and achieved an adjusted R^2^ of 0.22, *p* < 0.001. It was revealed that a 1‐year increase in the years of visits to diabetic clinic would increase the level of knowledge of diabetes patients by 2.28 keeping all other variables constant (*B* = 2.28, *p* = 0.002). Respondents on only insulin treatment modality would have an increased knowledge of diabetes by 4.17 (*B* = 4.17, *p* = 0.023) while those on combined therapy (both oral hypoglycaemic agent and insulin) would have an increased knowledge of diabetes by 7.26 (*B* = 7.26, *p* < 0.001) relative to those on oral hypoglycaemic agent (OHA) treatment modality, holding all other variables constant. Respondents without medically confirmed diabetic complications had an increase in knowledge of diabetes by 3.66 (*B* = 3.66, *p* = 0.002), keeping all other variables constant. Holding all other variables constant, respondents with no family history of diabetes and those with an unknown family history had a decreased knowledge of diabetes by 2.82 (*B* = −2.82, *p* = 0.023) and 3.04 (*B* = −3.04, *p* = 0.037), respectively, relative to respondents with a family history of diabetes.

**TABLE 3:** Multivariable regression analysis of knowledge as a dependent variable with clinical characteristics of participants

Variable	B1 ± SE	Beta2	*p* value	95% CI for B	
				Lower	Upper
Waist circumference	−0.03 ± 0.05	−0.04	0.449	−0.123	0.055
BMI	−0.10 ± 0.10	−0.06	0.288	−0.299	0.089
No. of years with diabetes	0.38 ± 0.80	0.04	0.631	−1.189	1.958
No. of years of visits to the diabetes clinic	2.28 ± 0.71	0.25	**0.002**	0.878	3.691
Type of treatment					
OHA	Ref				
Insulin	4.17 ± 1.83	0.12	**0.023**	0.568	7.777
Both OHA and insulin	7.26 ± 1.60	0.24	**<0.001**	4.110	10.408
Medically confirmed complication					
Yes	Ref				
No	3.66 ± 1.16	0.17	**0.002**	1.378	5.941
Not sure	4.43 ± 3.83	0.06	0.249	−3.111	11.971
Smoking					
Yes	Ref				
No	4.93 ± 2.53	0.10	0.053	−0.054	9.907
Family history					
Yes	Ref				
No	−2.82 ± 1.23	−0.13	**0.023**	−5.241	−0.392
Do not know	−3.04 ± 1.46	−0.11	**0.037**	−5.911	−0.178

Abbreviation: OHA, oral hypoglycaemic agent.

*Note*: Adjusted R^2^ = 0.22; overall model F test, *p* < 0.001.

### 3.4 Association between clinical characteristics and self‐care practice of participants

In Table 4, multivariable regression analysis revealed an adjusted R^2^ of 0.13, *p* < 0.001. It was revealed that keeping all other variables constant, a 1‐year increase in years of visits to the diabetic clinic would increase diabetes self‐care practice by 0.69 (*B* = 0.69, *p* = 0.005). Respondents on insulin treatment modality had a 40% increase in self‐care practice score (*B* = 1.4, *p* = 0.028) relative to respondents on oral hypoglycemic agents. Respondents without medically confirmed diabetic complications had 12% increased self‐care practice relative to respondents with medically confirmied diabetic complications (*B* = 1.12, *p* = 0.005). Self‐care practice in those with an unknown family history of diabetes decreased by 1.24 times (*B* = −1.24, *p* = 0.014) relative to participants with a family history of diabetes.

**TABLE 4:** Multivariable regression analysis of self‐care practice as a dependent variable with clinical characteristics of participants

Variable	B1 ± SE	Beta2	*p* value	95% CI for B	
				Lower	Upper
Waist circumference	0.01 ± 0.02	0.01	0.829	−0.027	0.034
BMI	−0.05 ± 0.03	−0.08	0.174	−0.112	0.020
No. of years with diabetes	−0.09 ± 0.27	−0.03	0.736	−0.633	0.447
No. of years of visits to the diabetes clinic	0.69 ± 0.24	0.23	**0.005**	0.208	1.167
Type of treatment					
Oral hypoglycemic	Ref				
Insulin	1.40 ± 0.63	0.12	**0.028**	0.155	2.639
Both oral and insulin	0.78 ± 0.56	0.08	0.161	−0.313	1.880
Medically confirmed complication					
Yes	Ref				
No	1.12 ± 0.39	0.16	**0.005**	0.344	1.897
Not sure	−2.09 ± 1.31	−0.09	0.112	−4.666	0.487
Smoking					
Yes	Ref				
No	0.88 ± 0.87	0.05	0.310	−0.825	2.589
Family history					
Yes	Ref				
No	−0.57 ± 0.42	−0.08	0.178	−1.394	0.260
Do not know	−1.24 ± 0.50	−0.14	**0.014**	−2.229	−0.256

*Note*: Adjusted R^2^ = 0.13, overall model *F* test, *p* < 0.001.

### 3.5 Association between self‐care practice and diabetes complications

In Table 5, the multivariable regression analysis show the relationship between self‐care practice and diabetes complications. It was revealed that having hypertension would decrease self‐care practice by 13% (*B* = −1.13, *p* = 0.021) while having diabetic foot would decrease self‐care practice by 4.46% (*B* = −4.46, *p* = 0.001), keeping all other variables constant.

**TABLE 5:** Multivariable regression analysis of self‐care practice as a dependent variable with diabetes complications among patients with type 2

Variable	B1 ± SE	Beta2	*p* value	95% CI for B	
				Lower	Upper
Retinopathy (Yes)	−0.75 ± 0.81	−0.05	0.359	−2.349	0.853
Neuropathy (Yes)	−1.06 ± 0.93	−0.06	0.253	−2.892	0.763
Nephropathy (Yes)	−0.10 ± 1.75	−0.01	0.955	−3.544	3.348
Cognitive impairment (Yes)	4.40 ± 3.48	0.07	0.206	−2.44	11.244
Heart disease (Yes)	−0.80 ± 1.57	−0.03	0.612	−3.888	2.292
Hypertension (Yes)	−1.13 ± 0.49	−0.13	**0.021**	−2.086	−0.169
Hypoactive sexual arousal (Yes)	0.31 ± 1.07	0.02	0.772	−1.803	2.425
Diabetic foot (Yes)	−4.46 ± 1.33	−0.18	**0.001**	−7.080	−1.831

*Note*: NB: the reference group for each of the variables in the model is the number of adjusted R^2^ = 0.03; overall model F test, *p* = 0.021.

## 4 DISCUSSION

In this study, the association of clinical factors with knowledge and self‐care practice among people with T2DM in Ghana has been established. It was observed that participants on insulin treatment modality, combined therapy (oral hypoglycaemic agent and insulin), participants without diabetic complication and participants without family history of diabetes had higher knowledge of diabetes.

Participants on combined treatment were found to have higher knowledge of diabetes compared with those on monotherapy. These patients might have had uncontrolled diabetes/comorbidities with monotherapy. This may result in more frequent contact with their healthcare providers. Healthcare workers would probably pay particular attention to these patients in terms of providing adequate education to enable them to achieve optimal glycaemic targets, thereby increasing their knowledge of the disease. The study showed that participants on insulin therapy and those without diabetic complications had higher self‐care practices. Consistent with a study done in Addis Ababa Ethiopia (Mamo & Demissie, 2016), our findings had shown that patients treated with insulin were more likely to have better self‐care practices compared with those on a tablet. The patients on insulin therapy probably had uncontrolled diabetes which may account for the relatively better self‐care practice. It is evident from this study that the patients using insulin had higher knowledge of diabetes compared to those who used only oral hypoglycaemic agents. Previous studies have demonstrated that knowledge of diabetes is positively correlated with self‐care practice (Karaoui et al., 2018). This means that the higher the knowledge of diabetes, the more likely participants are to perform self‐care. A contrasting result was found in a study done in Ethiopia where participants using combined therapy (insulin and oral hypoglycaemic treatment) were more likely to have better self‐care practices compared to their counterparts who used only oral medications (Abate et al., 2018).

Patients without diabetic complications were more likely to have good self‐care practices compared to those with complications. In this study, participants with diabetic foot and hypertension as complications had higher odds of poor self‐care practice. Patients with T2DM particularly with impaired vision, amputation or other complications may have challenges performing activities of daily living (Sparring et al., 2013) and may be hospitalized frequently which can affect their self‐care (Comino et al., 2015) than those without complications. To improve self‐care practice, there is a need for coordinated educational programs targeting patients with complications. Social support is required to improve self‐care practice among patients with complications. It is recommended that healthcare workers identify the specific needs of these patients and provide supportive care. Moreover, the findings of the present study show that participants without family history of diabetes had decreased self‐care practice. Having a family history of diabetes may provide patients with the opportunity to learn from other members who have had DM. A previous study found a family history to be associated with knowledge of diabetes (Kassahun et al., 2016). Information obtained from family members with DM could be very useful for effective self‐care practice.

According to a study done in Australia, BMI is associated with diabetes knowledge (Dixon et al., 2014). However, in the present study, there was no correlation between BMI and knowledge of diabetes or self‐care practice for the disease. The majority of participants in this study had lower educational status and may not know the health risks associated with obesity. Moreover, participants herein and of the Australian study may vary in their perception of the health implications of excess body weight. Overweight and obesity are usually seen as a mark of beauty and wealth in many developing countries including Ghana (Addo et al., 2009; Wahab et al., 2011) but mostly considered a health risk in several industrialized countries. Thus, the population of diabetes patients in the present study might not be conscious of the health implications of excess weight and may not take measures to control it and this has implications for targeted educational strategies by health providers.

### 4.1 Limitations of the study

The cross‐sectional design does not show temporal relationship or causality though it demonstrates associations between variables. The use of self‐report to measure adherence to self‐care practice can lead to recall and social desirability bias. Although social desirability bias could have occurred, the study identified factors that influence diabetes knowledge and self‐care practice which can be used to improve diabetes management. The convenience sampling procedure is less likely to produce an accurate and representative sample.

## 5 CONCLUSION

We have described several vital factors influencing diabetes knowledge and self‐care practice among people with T2DM in Ghana. Treatment modality and diabetic complications were significantly associated with both diabetes knowledge and self‐care practice. In particular, those who were on insulin and combined therapy (tablet and insulin) had higher knowledge and better self‐care practice. Self‐care was significantly influenced among those with, than those without diabetic foot and hypertension as complications. Thus, we recommend that health professionals providing care to people with diabetes evaluate these factors and provide appropriate education and support as well as training in self‐care especially for patients with diabetic complications.

## AUTHOR CONTRIBUTIONS

All authors contributed significantly to to the conception and design, collection of data, data analysis and interpretation. First author drafted the manuscript and all authors reviewed and provided their approval for publication of the final version.

## FUNDING INFORMATION

No funding was received for this study.

## CONFLICT OF INTEREST

The authors have no conflict of interest.

## ETHICAL STATEMENT

Approval for this study was granted by the Committee on Human Research, Publication and Ethics of the Kwame Nkrumah University of Science and Technology/Komfo Anokye Teaching Hospital (CHRPE/AP/576/18). Permission was sought from the heads of the hospitals to conduct the study in their facilities. The purpose and objectives of the study were explained to respondents and written informed consent obtained before data collection. The patients were informed that participation is voluntary and that they can opt‐out of the study at any time without any consequences to them. Respondents were assured of confidentiality and that no personal identifiers will be used in the questionnaire.

## Data Availability

The data that support the findings of this study are available on request from the corresponding author.
